# Preparation of Wood Fiber–Polyurethane Plastic Composite with Water Resistance and High Strength

**DOI:** 10.3390/ma18061314

**Published:** 2025-03-17

**Authors:** Xi Yuan, Shiyu Fu, Hao Liu

**Affiliations:** 1 State Key Laboratory of Pulp and Paper Engineering, South China University of Technology, Guangzhou 510640, China; ngwyxx@163.com (X.Y.); feliuh@scut.edu.cn (H.L.); 2 South China University of Technology-Zhuhai Institute of Modern Industrial Innovation, Zhuhai 519175, China

**Keywords:** eucalyptus sawdust, pretreatment, polyurethane prepolymer, composite, water resistance

## Abstract

The current widespread use of plastics is a significant source of environmental pollution and increases the carbon load in the atmosphere, which has precipitated an urgent drive to replace plastics with biomass-based materials. In this paper, we prepared a lignocellulose-based, high-strength, water-resistant composite based on eucalyptus waste sawdust combined with a polyurethane prepolymer. The preparation process included pretreating sawdust with deep eutectic solvents (DESs) to remove some of the lignin and hemicellulose. A prepolymer preparation involving isocyanate groups using the prepolymerization of polyethylene glycol (PEG) with hexamethylene diisocyanate (HDI) grafted the prepolymers to the hydroxyl of the pretreated wood fibers, which were subsequently blended with acetylated pretreated sawdust to create the composite. The composite contained 67% wood fibers, possessed good tensile strength, and exhibited Young’s moduli of 18 MPa and 484 MPa. It was water-resistant with a contact angle of 92° and had a low water absorption of 32%, and it maintained a wet tensile strength of 5.71 MPa. The composite offers several advantages, including UV protection and thermal stability. This high-performance wood waste composite provides an alternative green production option for producing plastic materials.

## 1. Introduction

The worldwide production and daily use of products made from petroleum-based plastics have resulted in widespread over-consumption of fossil fuel resources. In addition, disposing of such plastics has created a significant amount of white pollution, threatening human health [1]. Therefore, many countries are urgently developing green and renewable materials as substitutes for petroleum-based plastics [2,3]. Wood is rich in lignocellulosic fibers consisting of cellulose, hemicellulose, and lignin [4]. However, the inherent features of wood fibers include a porous structure and hydrophilicity, which limit their practical application as an alternative to plastics [5]. The intra- and inter-pores in the fibers of woody materials may not provide strength or flexibility in wood-based materials, so, in most cases, original woody materials cannot be processed in the same way as plastic. Hydrophilic woody fibers are not very compatible with hydrophobic polymers, which makes it difficult to produce high-quality water-resistant materials [6]. Many studies have focused on wood plastics made by blending wood and polymers [7]; these form the basis of the wood–plastic industry, which has grown to a capacity of over 2 million tons per year worldwide. In these products, wood powder is blended as a filler in a polymer matrix to reduce the proportion of plastic used. However, the plastics that remain in these products are not biodegradable. Fully degradable materials containing wood are attractive to researchers. In order to overcome the previously mentioned limitations of wood fibers, raw wood materials must be pretreated and modified during the preparation of wood-fiber-reinforced composites [8,9]. The aim of the treatment and modification is to easily modify the surface of the wood fibers and strengthen the composites. It is difficult for chemical reagents to access original wood or wood fibers because cellulose microfibrils adhere together through lignin and hemicellulose. The partial removal of lignin and hemicellulose from the wood is beneficial for subsequent modification. Among all treatment agents, deep eutectic solvents (DESs) are considered to be a greener and milder biomass pretreatment system [9]. Lignocellulosic material was pretreated with DESs to remove the lignin and hemicellulose, which improved the accessibility of the fibers [10,11] while maintaining the original structural strength of the lignocellulosic fibers. After DES treatment, aspen fibers can be converted to densified wood by hot pressing, achieving a tensile strength 800% higher than that of natural wood and 236% higher than that of compressed wood without delignification [10]. A molding process combining ball milling and DES treatment can produce woody bioplastics in which the remaining micro/nano-fibrillated cellulose is tied close together to form highly densified wood more than 10 times stiffer than natural aspen. Furthermore, when the regenerated nano-lignin on the fibrillated cellulose was exposed to liquids, the composite demonstrated improved hydrophobicity [12]. However, the micro- and nano-fibrillation process of wood fibers is an energy-intensive method, with high production costs, limiting their production on a commercial scale.

Cellulose derivatization is often adopted to change the surface properties of fibers so that the derivatized cellulose can be blended with hydrophobic polymers. The reagents for the cellulose derivatives include alkyl anhydrides, alkyl chlorides, and isocyanates [13]. Acetylated-modified cellulose fiber and isotactic polypropylene (iPP) can be blended very well in a twin screw because the hydroxyl groups on the surface of the cellulose fibers are acetylated and become compatible with hydrophobic polymers [14]. Cellulose fibrillated fibers covalently bond with the polyurethane molecular chain to form polyurethane elastomer composites, and the addition of 1% cellulose fiber allows a certain degree of phase separation to be maintained, increasing the thermal stability of the polyurethanes from 288 °C to about 300 °C [15].

In the present paper, strong, water-resistant wood composites were prepared using polyurethane prepolymer-reinforced wood fibers and hot pressing to take advantage of the high modulus of wood microfibrils modified with a polyurethane prepolymer to form strong, water-resistant wood composites. The wood fibers were pretreated with DES to improve their reactivity and accessibility and then combined with polyurethane prepolymers prepared using hexamethylene diisocyanate (HDI) and polyethylene glycol (PEG2000), which produced a dual-end isocyanate group (-NCO). The hydroxyl group on the surface of the pretreated wood fibers and the -NCO at both ends of the prepolymer were crosslinked under the hot-pressing method. Finally, the crosslinked wood fiber prepolymers were blended with pre-acetylated cellulose fibers to manufacture composites with a high biomass content and enhanced mechanical properties and water resistance. The present strategy can be used to prepare high-quality wood composites.

## 2. Materials and Methods

### 2.1. Materials

Eucalyptus wood powder (from Linyi, Shandong Province, China) was sieved through 100 mesh. Choline chloride (C_5_H_14_ClNO, 99%), L-lactic acid (C_3_H_6_O_3_, 80%), hexamethylene diisocyanate (HDI, 99%), concentrated sulfuric acid (H_2_SO_4_, 98%), acetic anhydride (C_4_H_6_O_3_, 98.5%) polyethylene glycol with molecular weight (Mw) of 400 (PEG 400) and 2000 (PEG 2000) g/mol, and N, N-dimethylformamide (DMF) (C_3_H_7_NO, 99.5%) were purchased from Shanghai Aladdin Biochemical Technology Co., Ltd., Shanghai, China. All chemicals were analytical reagents without further purification.

### 2.2. Wood Fiber Pretreatment and Preparation of Polyurethane Prepolymer

DES is a clear solution composed of choline chloride and L-lactic acid in a ratio of 1:9 (molar ratio) obtained by stirring at 60 °C. The eucalyptus-untreated wood powder fiber (UWF) was treated by DESs at 100 °C for 6 h with a solid–liquid ratio of 1:30 (weight (g) and volume (mL)). Then, the samples were transferred to a beaker and washed several times with deionized water (DI) to remove residual chemicals and lignin until the pH of the solution was reduced to 7 to obtain DES-pretreated wood fiber (DESF). The DESF (1.0 g) was reacted with acetic anhydride (15 mL) and catalyst H_2_SO_4_ (0.01 mL) at 80 °C under stirring conditions for 3 h. The acetylated DES-treated wood fiber (ADF) was washed with deionized water until neutrality was achieved and then dried in an oven.

PEG was reacted with HDI at NCO/OH = 2.0. HDI was placed in a three-necked flask under constant purging conditions with nitrogen. PEG was added dropwise to HDI under constant stirring at 70°. The obtained polyurethane prepolymer with the -NCO end group was stored in a closed container protected by nitrogen at −5 °C until use. The prepolymers created using PEG 2000 and PEG 400 were labeled as P2 and P4, respectively.

### 2.3. Reaction of Wood Fibers with Prepolymer

**Figure 1:**
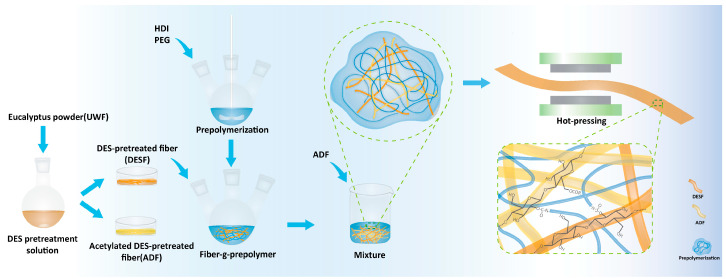
Flow chart for the preparation of polyurethane and wood fiber composite.

DESF (2 g), 40 mL of DMF, and a specific amount of prepolymer were added to a 250 mL flask, subjected to a grafting reaction under a nitrogen atmosphere, and underwent stirring at 100 °C for 10 h. The products were poured onto a Teflon mold and dried in an oven at 80 °C for 8 h. The products with a ratio of DESF to prepolymer (from 1:3 to 1:5) are named DESFP2-13 and DESFP2-15. The procedure used is depicted in Figure 1, including wood fiber treatment with DES, prepolymerization of PU grafted to wood fiber, and composite formation.

### 2.4. Preparation of Prepolymers–ADF Composite

ADF and lignocellulose-g-prepolymer are blended well in mass ratios of 1:4, 3:4, and 5:4, followed by 60 min of hot pressing at 110 °C and 10 MPa. The preparation process is presented in detail in Figure 1. The composites based on PEG 2000 and PEG 400 are named ALP2-1, ALP2-3, ALP2-5, ALP4-1, ALP4-3, and ALP4-5, based on the different ADF ratios involved (Table 1).

Prepolymer-g-fiber is the product of the reaction between prepolymer and DESF in the previous step; the ratio of DESF to prepolymer is 1:3 (i.e., the content is four parts of the total).

### 2.5. Characterization and Testing

#### 2.5.1. Scanning Electron Microscopy (SEM)

The surface and cross-section morphology of P2, DESFP2-13, ALP2-1, ALP2-3, and ALP2-5 films were observed on SEM (JSM JOEL, Tokyo, Japan SU5000) at an accelerating voltage of 30 kV. All specimens were coated with a thin layer of gold prior to observation.

#### 2.5.2. Attenuated Total Reflectance Infrared Spectroscopy (ATR-IR)

The infrared spectra of all the samples were acquired using a Nicolet IS50 FT-IR spectrometer (THERMO Nicolet, Waltham, MA, USA) equipped with an attenuated total reflection (ATR) accessory. Sixteen scans were performed in the range of 650~4000 cm^−1^.

#### 2.5.3. Thermal Gravimetric Analysis (TGA)

TGA tests of ALP-x and DESFP-x were carried out using a Thermogravimetric Analyzer Model TGA 2 (Mettler Toledo, Greifensee, Switzerland). Each sample was heated from 30 °C to 800 °C in a nitrogen atmosphere at a rate of 20 °C min^−1^. The temperature at the intersection of the points at which the sample loses 20% and 50% of its weight and intersects the baseline extension was identified as the decomposition temperature during the thermal stability evaluation.

#### 2.5.4. Ultraviolet–Visible–Near Infrared Spectroscopy (UV–Vis-NIR)

The transmission spectra of the films in the wavelength range of 200~800 nm were measured on a UV-2600i spectrophotometer (Shimadzu Instrument (Suzhou) Co., Ltd., Suzhou, Jiangsu, China) to determine the UV-blocking properties of the films. Prior to the analysis, all samples were cut into 4 cm × 2 cm rectangles and vacuum-dried at 50 °C for 12 h to remove any absorbed moisture.

#### 2.5.5. Elemental Analysis

The specimens were dried and a Vario EL cube elemental analyzer was used to determine the amount of N, C, and H elements within each sample.

#### 2.5.6. Water Stability Tests

The static water contact angle (WCA) of the films was evaluated at room temperature using a contact angle meter (Biolin T200-Auto3 Plus, Gothenburg, Sweden). The contact angle of the films was measured using a camera. In addition, the prepolymer and wood fiber composite films were submerged in water and, after excess water was removed from the surface of the sample using filter paper, the weight of the samples was measured on a calibrated balance. The water absorption of the samples was measured every 10 minutes until it stabilized. Water stability tests were conducted by submerging the prepolymer membrane and wood fiber composite membrane in water for 30 days.

#### 2.5.7. Tensile Test

The tensile strength of the films was determined using a tensile tester (INSTRON, Norwood, MA, United States). The specimens were stretched along the length of the specimen until they ruptured; this took place at room temperature and at a constant test speed of 10 mm/min. The samples were completely immersed in deionized water for 2 h and both the wet strength and dry strength were determined. Excess water was wiped off the film prior to determining the wet strength.

## 3. Results and Discussion

### 3.1. Pretreatment of Wood Fibers for Modification

**Figure 2:**
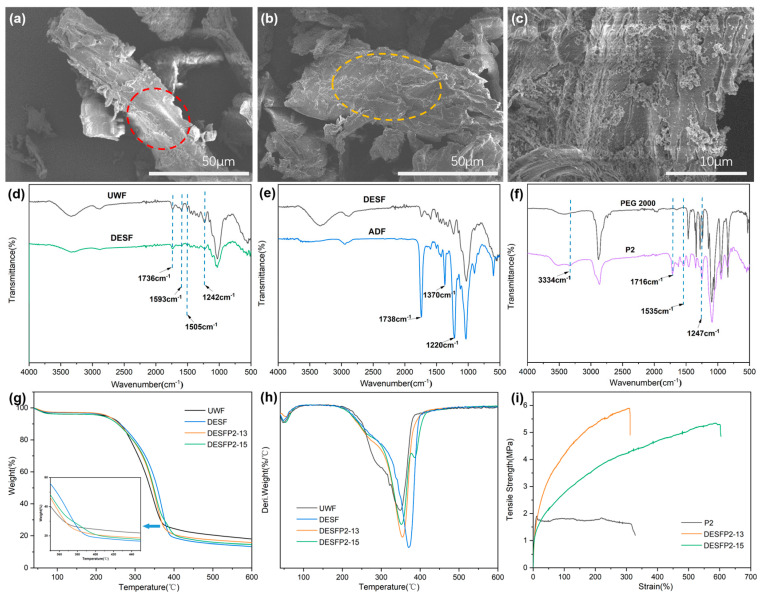
SEM images: (**a**) UWF, (**b**) DESF, and (**c**) ADF; ATR-IR spectra: (**d**) UWF and DESF, (**e**) DESF and ADF, and (**f**) polyethylene glycol 2000 (PEG 2000) and polyurethane prepolymers (P2); (**g**) TGA plots of UWF, DESF, DESFP2-13, and DESFP2-15; (**h**) DTG plots of UWF, DESF, DESFP2-13, and DESFP2-15; (**i**) tensile test plots of P2, DESFP2-13, and DESFP2-15.

Wood fibers were treated with DES prior to the grafting reaction; the morphologies of the wood fibers, grafted fibers, and acetylated fibers are shown in Figure 2. The surface of untreated eucalyptus wood powder (UWF) in Figure 2a is relatively smooth and tight (indicated by a circle in Figure 2a) and aggregated with small fragments. After DES pretreatment, the fiber surface roughness increased (as shown in the circle in Figure 2b) due to the redistribution and rearrangement of the dissolved lignin from the DES pretreatment liquor [16]. The difference between untreated wood powder and DES pretreated fibers (DESFs) could be determined based on their IR spectroscopy. The absorption peaks at 3438 and 2925 cm^−1^ in Figure 2d correspond to the -OH and -CH_2_ scaling of cellulose. After DES treatment, the DESFs showed no new characteristic peaks compared to untreated wood fibers, but the intensity of some peaks attenuated, such as peaks at 1736 cm^−1^ corresponding to C=O stretching vibration in ester linkages on hemicellulose, 1505 and 1593 cm^−1^ to C=C stretching vibration of the aromatic ring in lignin, and 1242 cm^−1^ to C-O stretching vibrations in lignin and hemicellulose, suggesting that partial removal of lignin and hemicellulose from wood fibers is achieved during the treatment process [17].

DES treatment also affected the thermal stability of wood fibers. The TGA and DTG curves of UWF and DESF are shown in Figure 2g. At temperatures between 40 °C and 105 °C, all samples exhibited slight mass losses, which could mainly be attributed to the evaporation of adsorbed water. Hemicellulose and cellulose generally decompose at temperatures between 190 and 380 °C [18], while lignin decomposes more slowly than cellulose and hemicellulose at temperatures between 150 °C and 900 °C [19]. The residual content of UWF at 500 °C was 20.43%, which was higher than the residual content of 15.06% after DES pretreatment. This is because the untreated wood powder contains greater amounts of lignin, and more char carbon remains in residuals after thermal decomposition. Partial removal of lignin and hemicellulose may reduce the presence of residues identified via TGA measurements following DES pretreatment.

Wood microfiber is renowned for its strength and high elastic modulus. After DES treatment, the microfiber residues retain some of the removed lignin and hemicellulose. Ultra-strong, densified wood which has been pressed and had all of its lignin and hemicellulose removed [20] can even withstand the impact of an air-gun bullet. However, alternatives to plastic need to have similar processibility to actual plastic. In this research, prepolymers made from polyurethane of HDI and PEG were prepared. These prepolymers contained two end isocyanate groups, which can react with the hydroxyl groups on the surface of microfibers that have undergone DES treatment. The infrared spectra of the prepolymer (P2) and PEG2000 are depicted in Figure 2f. The newly formed peak at 1717 cm^−1^ in the infrared spectrum of P2 is caused by the C=O stretching vibration of the carbamate group. The N-H stretching vibrations of the amide groups in the product correspond to peaks at 3334 cm^−1^ and 1535 cm^−1^. The peaks at around 1246 cm^−1^ are attributed to asymmetric C-O-C stretching [21,22]. Since unreacted HDI was removed from the system, the formation of polyurethane prepolymers was achieved as described above.

Subsequently, the DESFs were reacted with the prepared prepolymer, and the products were washed with ethanol/water and extracted with acetone to obtain the DESF-grafted product. DES pretreatment can improve the accessibility of the exposed -OH group on wood microfibers, allowing them to react with the isocyanate (-NCO) groups in P2 [23]. This is supported by elemental measurements of products. The elemental analysis data in Table 2 reveal that, following a grafting reaction between DESF and P2 in unreacted wood fiber, the N content increased from 0.03% to 0.3% in DESFP2-13 and to 0.51% in DESFP2-15, and this was accompanied by an increase in hydrogen (H) content (DESF: 5.738%, DESFP2-13: 6.543%, and DESFP2-15: 6.682%), which demonstrated the reaction of -NCO groups in prepolymers with hydroxyl of wood fibers. Because of the grafting reaction, the carbon content also increased a little in other products in comparison to DESF.

Moreover, the thermogravimetric analysis in Figure 2g,h also supported a link between P2 and cellulose fibers. Two major decomposition stages can be observed on the DTG curves of the DESFLP2-15 in Figure 2h, based on the reaction of DESF and P2 in a ratio of 1:5. The peaks between 230 °C and 370 °C are attributed to the decomposition of the carbamate, the PEG fraction, and the cellulose backbone. The hump peak from 370 °C to 470 °C represents the breakdown of the aliphatic chain in the PU side chain [24,25], demonstrating that P2 were successfully grafted to cellulose fibers. These findings suggest that there is covalent bonding between the isocyanate and DESF, which improves the interaction and adhesion between the two substances and may contribute to an increase in mechanical strength, as shown in Figure 2i.

The reaction conducted in heterogeneous systems and the reactivity of wood powders may depend on the size of the particles involved. In general, non-fibrillated wood fibers have less successful reactions than small-size nanocellulose. Adjusting the ratio of wood fiber and prepolymers may enhance the grafting process, influencing the mechanical strength of products. In Figure 2i, DESFLP2-13 with a reaction ratio of 1:3 was selected for subsequent experiments. Although its plastic and lignocellulose modification was not as effective as that of DESFLP2-15, it had a higher tensile strength, and it was selected for future tests in light of its compatibility, film strength, and effective use of lignocellulose, as well as its ability to maintain a certain degree of cross-linking.

Finally, adding an excessive amount of UWF to the prepolymer during pretrials led to agglomeration. We tentatively blended acetylated lignocellulosic fibers with the composites to ensure better interfacial compatibility; the hydroxyl groups on the cellulose molecules of the surface of fibers were grafted with hydrophobic groups (e.g., the acetyl group) to increase the hydrophobicity and plasticity of the lignocellulosic fibers [26,27]. In this case, acetyl groups were used as substitutes for hydroxyl groups on the surface of wood fibers, and the hydrogen bonding network in lignocellulose was destroyed, potentially improving its compatibility with polyurethane. Acetylation also changes the morphology of wood fibers, making them more accessible and improving subsequent reactions [28,29,30]. After the acetylation reaction, the acetylated DES-treated wood fiber (ADF) showed more pronounced infrared absorption near 1738 cm^−1^ and 1370 cm^−1^ than DESF (see Figure 2e), corresponding to the stretching vibration of C=O and the wagging vibration of -CH_3_ in acetyl groups. This suggests that surface carbohydrates in the DESF were successfully grafted with acetyl groups. Acetylation also alters the surface state of the wood fibers. Some aggregated acetylated microfibers can be observed on the surface of ADF in Figure 2c, as the microfibrillar structure of the fibers was destroyed during acetylation. Consequently, the surface roughness of the fibers increased dramatically after treatment with acetic anhydride [31].

### 3.2. Mechanical Properties of Composite Films

**Figure 3:**
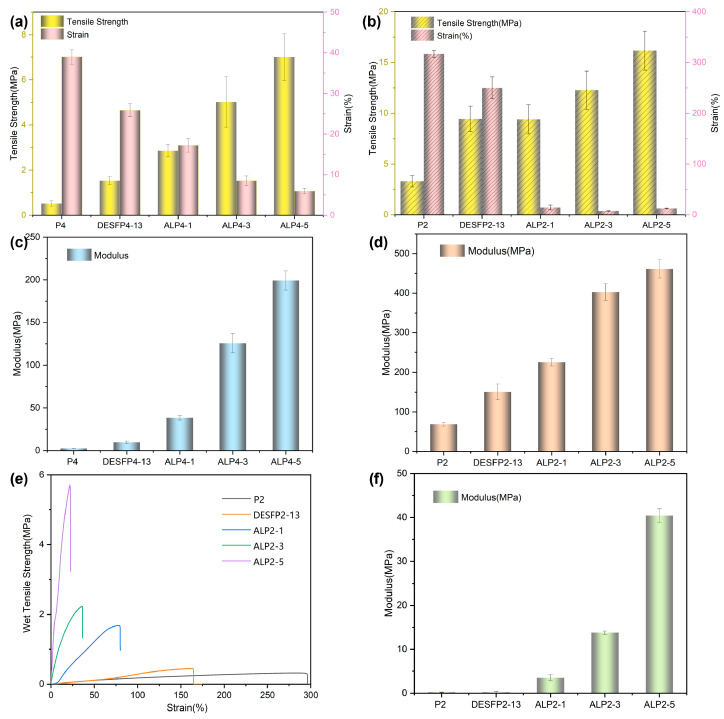
Polyurethane prepolymers and composite films prepared with different PEG chain lengths: (**a**,**b**) tensile strength and elongation at break; (**c**,**d**) Young’s modulus of P4, DESFP4-13, ALP4-1, ALP4-3, and ALP4-5 and P2, DESFP2-13, ALP2-1, ALP2-3, and ALP2-5; (**e**,**f**) wet tensile test plots and Young’s modulus of P2, DESFP2-13, ALP2-1, ALP2-3, and ALP2-5.

The chain length of polyol PEG in prepolymer may affect the mechanical properties of the composites. Two substances (PEG400 and PEG2000) with different chain lengths were chosen as soft chain segments for prepolymer preparation, as these segments confer elastomeric properties to prepolymer films [32]. An increase in the molecular weight of the polyol increases the microscopic phase segregation, crystallinity, and disordered structures in the soft and hard domains in the PU matrix [33,34]. In contrast, when low-molecular-weight polyols are utilized, more urethane bonds and hydrogen bonds form in the synthesized polyurethane prepolymers, increasing the rigidity of the film, and their lower crystallinity reduces the tensile strength. The flexibility and strength of the original prepolymer films, P4 and P2, are shown in Figure 3a,b. The tensile strength of P2 (3.30 MPa) is superior to that of P4 (0.52 MPa). Subsequently, the DESF reacted with the prepolymers to form prepolymer-grafted fibers. The strength of prepolymer-grafted fibers increased significantly, with their tensile strength increasing from 0.52 MPa to 1.53 MPa for DESFP4-13 and 3.30 MPa to 9.46 MPa for DESFP2-13. When the terminal hydroxyl group of PEG is converted to a reactive functional group, it can be used as part of a junction [35]. Therefore, isocyanate (-NCO)-capped PEG tends to produce PEG-isocyanate. When the prepolymers react with the hydroxyl groups on the surface of treated fibers, a stable carbamate bond is formed [36]. Low-molecular-weight (Mw) PEG-isocyanate with a high ratio of -NCO content per unit mass can create more carbamate covalent bonds between fibers and prepolymers compared to the high Mw PEG-isocyanate. Additionally, we found that the composites have a higher degree of chain entanglement and were unable to crystallize, which reduced their tensile strength. The product with low Mw PEG was more rigid and brittle, which is not favorable for practical applications. Overall, considering the prepolymer reactions and their composite properties, PEG2000 is the most favorable polyurethane material.

Prepolymer-grafted fibers have wood content of less than 50%, meaning that additional wood fibers should be added for composites formation. Raw wood fibers and DES-treated fibers (DESFs) have poor compatibility with the prepolymers, so the treated wood fibers are acetylized to ADFs with more surface hydrophobicity. After the addition of ADF (Table 1), the composites in various ratios of ADF and fiber-g-prepolymers showed increases in their tensile strength and Young’s modulus. The tensile strength (Figure 3a,b) and Young’s modulus (Figure 3c,d) of the composites (ALP4-x or ALP2-x) increased significantly with the increase in blended ADF compared with P4 or P2 and DESFP4-13 or DESFP2-13. The PEG 400 prepolymer and subsequent composites are not as good as PEG 2000 in terms of their strength and Young’s modulus. The composite containing approximately 67% wood filler showed a Young’s modulus (ALP2-5) of 484 MPa and tensile strength of 18 MPa, 7 times and 5.5 times those of P2. However, the elongation of products at break was greatly reduced. The elongation at break of ALP2-5 was only 12.6% of P2, and the same was observed for P4 and its composites. In fact, raw wood powder is difficult to uniformly disperse in a hydrophobic polymer matrix. Acetylation of wood fibers reduces the hydrogen bonding between cellulose molecules or fibrils by replacing some of the hydroxyl groups on the surface of the fibers. The remaining hydroxyl groups of ADF continue to participate in a cross-linking reaction with -NCO, which contributes to the formation of mesh structure in composites, enhancing their mechanical properties. In polyurethane materials, the crystallinity of components in hard segments may affect the mechanical properties of materials, and the chain entanglement contributes to elongation [37,38,39]. However, the high amount of wood fiber in the matrix results in a high ratio of hard segment chains, enhancing the rigidity of the material while reducing the elongation of the composite at break. For wood fiber composites using polyurethane as matrix, the portion of wood fibers was generally low in the final composites, although the composites in the scheme of this paper exhibited enhanced mechanical properties and significant increases in biomass content. Compared to wood fiber composites based on high-strength plastics, such as polyethylene terephthalate (PET) and high-density polyethylene (HDPE) [40,41,42], the approach described in this paper maintains a high proportion of wood fiber but does not involve additional plasticizer and coupling agents, improving the composite’s biocompatibility.

The strength in the moisture atmosphere is significant for material application. The wet tensile strength and Young’s modulus were tested as shown in Figure 3e,f. Composites made with ADFs blended with hydrophobic surface groups show improved strength in water. Both the wet tensile strength and Young’s modulus improved as the amount of ADF in the composites increased. The wet tensile strengths of P2 and DESFP2-13 in Figure 3e were only 0.32 and 0.46 MPa; however, they increased to 1.69, 2.24, and 5.71 MPa for ALP2-1, ALP2-3, and ALP2-5, respectively. The Young’s moduli of ALP2-1, ALP2-3, and ALP2-5 increased to 3.51, 13.8, and 40.4 MPa, much higher than those of P2 (0.22 MPa) and DESFP2-13 (0.24 MPa), as shown in Figure 3f. In addition, the elongation at break of the composites after a set period of immersion in water also improved compared to that of dried samples, and this was attributed to the ADF content. The elongation at break of ALP2-1, ALP2-3, and ALP2-5 increased from 14%, 7.5%, and 13% in dry tests to 79%, 36%, and 22% in wet tests, respectively. The improved elongation of the composites may have been the result of some water molecules entering the materials to enhance the lubrication of fibers at the expense of the materials’ strength. Regardless, the water absorption can be maintained at a low constant so that the composites can retain their strength whilst also improving their elongation, which will allow them to provide a certain level of flexibility.

In contrast, the elongation at break of P2 and DESFP2-13 decreased from 316% and 250% in dry conditions to 291% and 163% in wet conditions. This is because too many water molecules entered the materials, potentially decreasing the interaction forces among the molecule chains. The polyurethane prepolymer (P2) and DESFP2-13 immersed in water for 30 days became very weak and inelastic (no supporting data are available), while the composite-blended ADF was effectively prevented from dissolving in water and maintained a good level of mechanical strength. The ADF contained residual lignin, which continued to interact with active groups on P2 motif of DESFP2-13 and bonded more successfully with the composite, similarly to the composite adhered to by lignin and wood adhesives, maintaining good mechanical properties and excellent water stability [43].

### 3.3. Fracture Morphology of Composite Films

**Figure 4:**
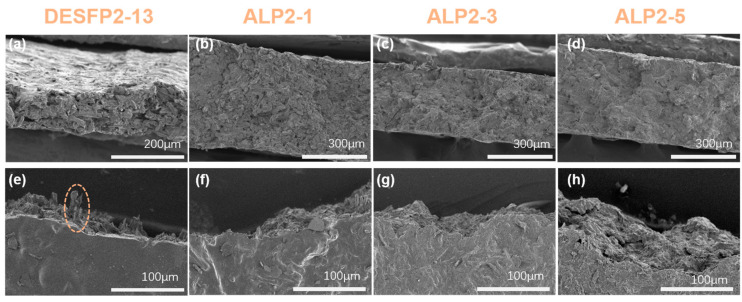
SEM images of fractures in composites containing different amounts of wood fiber. (**a**–**d**) Fractures in cross-section; (**e**–**h**) fractures in parallel.

The good binding of the constituents in the composite can be recognized based on the way the composite fractured during stretching, as shown in Figure 4. The fracture of DESFP2-13 films derived from DESF-grafted P2 exhibits a loose structure in the cross-section (see Figure 4a), and the fracture in parallel possesses some drawn-out fibers (circled in Figure 4e). This is because DESFP2-13 is composed of DESFs linked with highly stretchable P2, so that, when DESFP2-13 film is stretched, the fibers can be drawn out from the film, while the inner section of the films remains loose, as shown in Figure 4a. After the introduction of ADF and hot pressing, the flexible polyurethane prepolymers wrapped around and covered the fibers to form composites with denser stacking (see Figure 4b–d), which indicates that the gaps between the fibers in the network were compressed after hot pressing [44]. The fracture section in the parallel parts of the films (Figure 4e–h) shows that the DESFP2-13 film cross-section is broken by a loose fiber mesh through macroscopic fiber slippage, and the drawn wood fibers are clearly visible at the cross-section in Figure 4e. Meanwhile, ALP2-1, ALP2-3, and ALP2-5 in Figure 4f–h exhibit a tight laminar structure with stacked wood fibers, which is in agreement with that depicted in the cross-sections. Acetyl groups on the surface of DESF confer their hydrophobic surface properties due to the hydroxyl groups converting to hydrophobic acetyl group improving the compatibility between wood fiber (ADF) and polyurethane prepolymer grafted to wood fiber (DESFP2). The wood fibers also act as reinforcing agents and fillers in the composite, strengthen the mechanical properties of the original matrix, improve its hydrophobicity, and increase the amount of biomass material that is added [14]. On the other hand, the invisibility of single fibers also suggests that the reticulated polyurethane prepolymer structure binds the fibers and, due to the covalent bonding between the wood fibers and the -NCO jointed prepolymers, the addition of the polyurethane prepolymers fills the empty areas around the wood fiber molecules; thus, the matrix and wood fibers successfully coexist to improve the formation of the composite [45]. However, the excessive addition of wood fiber makes it difficult for the polyurethane prepolymer to completely cover it. At the same time, a serious agglomeration phenomenon occurs, destroying the interfacial bonding between wood fiber and polyurethane prepolymer. This means that the ADF cannot be uniformly distributed in the polyurethane matrix, leading to a higher concentration of stress and a decline in composite materials’ mechanical properties [46,47]. In the actual experiments, compared with ALP2-5, the addition of a higher proportion of ADF causes the film to break after hot pressing, resulting in a reduction in the overall performance.

### 3.4. Thermal Properties of Composite Films

**Figure 5:**
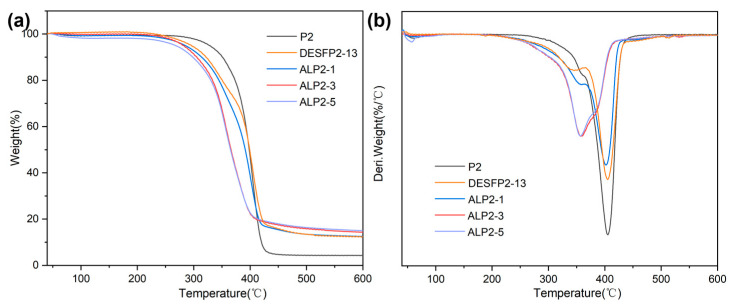
(**a**) TG and (**b**) DTG curves of P2, DESFP2-13, ALP2-1, ALP2-3, and ALP2-5.

The TG and DTG curves of the films are used to demonstrate their thermal properties in Figure 5a,b. The weight loss in the range of 40–105 °C is related to the evaporation of absorbed water in P2 in the TG plot. The first decomposition region between 250 and 370 °C is attributed to the degradation of the carbamate groups, while the second region between 370 and 470 °C is attributed to polyol degradation in the film [15]. The presence of wood fibers in the composite increases the residues present in the thermal degradation test. The amount of DESFP2-13, ALP2-1, ALP2-3, and ALP2-5 residue at 500 °C increased to 13.5%, 13.5%, 15.8%, and 16.4% compared to the 4.31% identified in P2, as shown in Figure 5a. Wood fibers merged in composites caused all the composites to decompose earlier in the TGA test. The initial decomposition temperatures of DESFP2-13 shifted to 315 °C from 357 °C in P2 and, as more ADF was added, the initial decomposition temperatures of ALP2-1, ALP2-3, and ALP2-5 increased to 311, 312, and 309 °C because of the existence of partial residual hemicellulose in the wood fibers, as this substance degrades at around 240 °C [48,49]. All the composites remained thermally stable at 300 °C, which indicates that the materials can be thermally processed at this temperature. In all, after DES treatment and grafting with polyurethane prepolymer, wood fibers can be thermally processed when blended with acetylated fibers without any plasticizers.

The DTG of DESFLP2-13 and ALP2-1 in Figure 5b shows a similar heat-loss profile to that of P2, with two characteristic decomposition phases dominated by polyurethane. For ALP2-3 and ALP2-5, the T_max_ of the first decomposition stage is around 350 °C and the T_max_ peak of the second stage (400 °C for P2) gradually diminishes and shifts towards the T_max_ of UWF (387 °C, in Figure 2f). These results indicate that modified wood fibers may form strong covalent bonds in the composites and exhibit cross-linking in the polyurethane prepolymer matrix. The prepolymer polyurethane grafted to fibers provides good compatibility, which allows wood fibers to be evenly distributed throughout the prepolymer film. In addition, the thermal stability of polyurethane composites varies with the composition ratio, with two peaks in DTG corresponding to the thermal characteristics of the two original components. Therefore, when too much ADF is added, the onset of decomposition temperature, T_max_, and residual content appears between that of wood fibers and polyurethanes, meaning that the thermal decomposition is dominated by high wood fiber content.

### 3.5. Water Resistance of Composite Films

**Figure 6:**
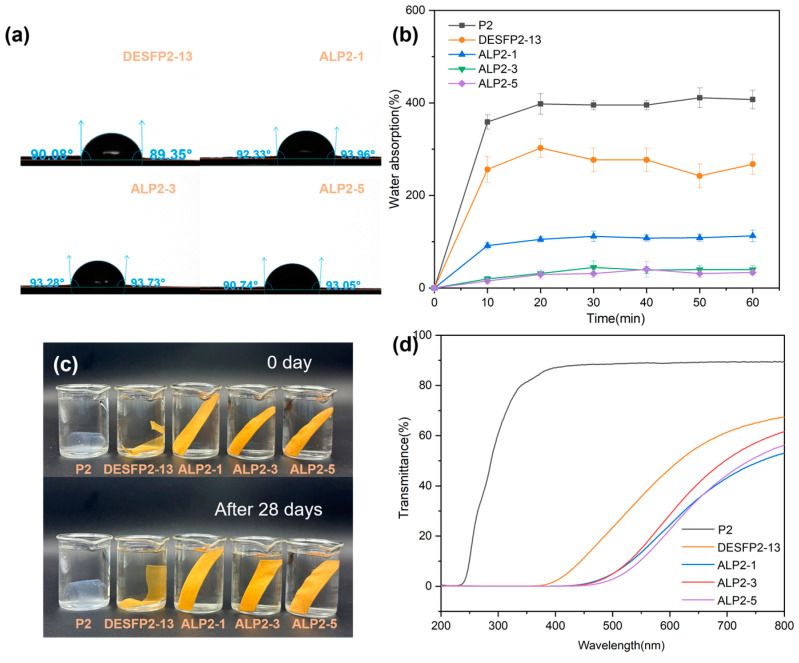
(**a**) Water contact angle; (**b**) water absorption (P2, DESFP2-13, ALP2-1, ALP2-3, and ALP2-5); (**c**) water stability test of the film; (**d**) UV transmittance.

The contact angle (CA) of water on the surface of the sample is used to demonstrate the material’s hydrophobicity or hydrophilicity. It should be greater than or equal to 90° for hydrophobic materials [50]. In general, hydrophobic materials have low water absorption, which helps them to maintain their strength in wet conditions. As shown in Figure 6a, all the wood fiber composites had contact angles of approximately 90° (DESFP2-13: 90°, ALP2-1: 93°, ALP2-3: 94°, and ALP2-5: 92°), which indicates that the addition of ADF improved the surface hydrophobicity of the polyurethane prepolymer. The rise in the CA is attributed to the increased interaction between the prepolymer and the DESF through polyurethane, which reduced the likelihood of the wood fibers in the membrane being exposed to water. The presence of lignin in the wood fibers (DESFs) after pretreatment also increased the hydrophobicity of the membrane [43]. More importantly, acetylation of the hydroxyl groups on the surface of the wood fiber converted the hydrophilicity of fiber surface to hydrophobicity, meaning that composites with higher content of ADF possessed higher hydrophobicity and water stability.

The water absorption curves in Figure 6b show that water absorption occurred rapidly when the materials were initially submerged in water, and the absorption rate of all the samples increased with the immersion time but at different levels. The water absorption of DESFP2-13, ALP2-1, ALP2-3, and ALP2-5 at 10 min was 256%, 92%, 20%, and 15%, respectively, whereas that of P2 was 359%. The water absorption of composites decreased with the addition of acetylated wood fiber. The water absorption rate rose slightly at 20 min, and no further increase was observed after 20 min. The water absorption rate of ALP2-5 after saturation was about 32%, much lower than that of P2 (about 400%). The saturated composites absorbed very little water, even after being immersed in water for a few months. This is because there is a conjunction between the polyurethane prepolymer and the wood fibers, combined with the hydrophobic acetylated wood fiber filling reducing the porous structure formed by the interlacing of the wood fiber network, which may allow for a relatively small amount of air trapping or voids within the composite [51,52]. When the wood fiber content in the composites increased, especially the acetylated wood fibers, the water struggled to diffuse into the polymer molecular chains and interstitial spaces at the fiber/matrix interface, attenuating water absorption and swelling of the material [53]. As such, our material is suitable for outdoor and load-bearing applications.

### 3.6. UV Resistance of Composite Films

The UV resistance of films is important when creating packaging materials for fragile products, such as cosmetics and pharmaceuticals. In experiments, the materials’ transmittance was measured in the UV light range. In this range, the transmittance will be low if the UV light is absorbed by the materials. The transmittance of films containing various amounts of wood fibers is shown in Figure 6d. The transmittance of prepolymer polyurethane P2 was 89%, while it was 36% for DESFP2-13 with 25 wt% wood fibers, which means the films became less transparent. The transparency of the films decreased further as ADF was blended with the composite. The transmittance of a composite (ALP2-5) containing 67% wood fibers decreased to 10.53%. This is because an increase in the wood fiber content in the film led to an increase in light absorption [54]. The pure synthesized prepolymer P2 has high transparency, but low natural UV absorption in the range of wavelength 190–400 nm (Figure 6d). The film made of wood fibers grafted with P2 (DESFP2-13) showed good UV absorbance because the DESFs still contained residual lignin after the DES treatment. ALP2-1, ALP2-3, and ALP2-5 contain more DESF, which allows them to absorb almost all UV light and they could potentially be used as UV-blocking materials because their aromatic structure and the large number of phenols, ketones, and intramolecular hydrogen bonds that the residual lignin possesses grant these composites excellent UV absorption properties similar to those of other lignin-containing composite films [55,56,57]. In addition, the composites containing suitable amounts of ADF can reduce transmission of visible blue light in the 400–500 nm range (high-energy light), while the composite containing a large amount of ADF shows almost no light transmission. In general, visible high-energy blue light at a level of 400–500 nm affects plant photosynthesis and can cause damage to the retina and the skin [58], which suggests the prepared film could potentially be used to provide ground cover in agricultural applications and may have use as an anti-blue-light polymeric material.

## 4. Conclusions

In this study, polyurethane prepolymers were prepared by reacting PEG with HDI, and the synthesized prepolymers were reacted with DESF to form prepolymer-grafted DESFs. Subsequently, acetylated DESFs (ADFs) were blended in to achieve compatibility and boost the hydrophobicity of wood-fiber-based composites (ALPs), which have good mechanical properties and water stability. The tensile strength and Young’s modulus of ALP2-5 composed of 67% woody fibers were 18 MPa and 484 MPa, respectively. All the composite films in this study had a contact angle of over 90°, and the water absorption of ALP2-5 was only 32% after complete water swelling, much lower than that of pure polyurethane prepolymers (400%). At the same time, the composite film exhibited UV absorption properties. In summary, the composite materials prepared using this strategy and with high wood fiber content can be hot-processed without the need for plasticizers while maintaining high tensile strength, Young’s modulus, water stability, thermal stability, and UV protection. This provides a route for manufacturing bioplastic from lignocellulose resources and contributes to research on green materials that can be used as alternative, eco-friendly packaging. In future research, it may be possible to select various types of isocyanates, polyols, and chain extenders as suitable raw materials that can be used to create excellent composites with further enhanced properties. We hope to see further improvements in the characteristics of different species of wood and further examination of the effect of different wood fiber pretreatment methods on the performance of composites. As composite materials containing wood fiber continue to improve, we expect that they will become increasingly widespread as a replacement material in the plastic matrix.

## Figures and Tables

**Table 1:** Constituents of prepared composites.

PEG in Prepolymer	ADF and Prepolymer-g-Fiber	Composites
PEG 2000	Without ADF and DESF	P2
PEG 2000	1:4	ALP2-1
PEG 2000	3:4	ALP2-3
PEG 2000	5:4	ALP2-5
PEG 400	Without ADF and DESF	P4
PEG 400	1:4	ALP4-1
PEG 400	3:4	ALP4-3
PEG 400	5:4	ALP4-5

**Table 2:** Elemental analysis of DESF, DESFP2-13, and DESFP2-15.

Scheme	N (%)	C (%)	H (%)
DESF	0.03	49.06	5.738
DESFP2-13	0.30	49.13	6.543
DESFP2-15	0.51	49.14	6.682

## Data Availability

The original contributions presented in the study are included in the article, further inquiries can be directed to the corresponding author.

## References

[1] Rosenboom J.-G., Langer R., Traverso G. (2022). Bioplastics for a circular economy. Nat. Rev. Mater..

[2] Dong L., Cao G., Zhao L., Liu B., Ren N. (2018). Alkali/urea pretreatment of rice straw at low temperature for enhanced biological hydrogen production. Bioresour. Technol..

[3] Jagannathan P., Muthukumaran C., Tamilarasan K. (2017). A sequential pretreatment of lignocelluloses in bamboo biomass to fermentable sugars by acid/enzymatic hydrolysis. 3 Biotech.

[4] Xia Q., Chen C., Yao Y., Li J., He S., Zhou Y., Li T., Pan X., Yao Y., Hu L. (2021). A strong, biodegradable and recyclable lignocellulosic bioplastic. Nat. Sustain..

[5] Li P., Yang C., Jiang Z., Jin Y., Wu W. (2023). Lignocellulose pretreatment by deep eutectic solvents and related technologies: A review. J. Bioresour. Bioprod..

[6] Sreekala M.S., Kumaran M.G., Joseph S., Jacob M., Thomas S. (2000). Oil palm fibre reinforced phenol formaldehyde composites: Influence of fibre surface modifications on the mechanical performance. Appl. Compos. Mater..

[7] Nassar M.M.A., Alzebdeh K.I., Pervez T., Al-Hinai N., Munam A. (2021). Progress and challenges in sustainability, compatibility, and production of eco-composites: A state-of-art review. J. Appl. Polym. Sci..

[8] Xu J., Xu J., Zhang S., Xia J., Liu X., Chu X., Duan J., Li X. (2018). Synergistic effects of metal salt and ionic liquid on the pretreatment of sugarcane bagasse for enhanced enzymatic hydrolysis. Bioresour. Technol..

[9] Alvarez-Vasco C., Ma R., Quintero M., Guo M., Geleynse S., Ramasamy K.K., Wolcott M., Zhang X. (2016). Unique low-molecular-weight lignin with high purity extracted from wood by deep eutectic solvents (DES): A source of lignin for valorization. Green Chem..

[10] Fan Z., Sun H., Zhang L., Zhao X., Hu Y. (2022). Lightweight, High-Strength Wood Prepared by Deep Eutectic Solvent Treatment as a Green Structural Material. ACS Sustain. Chem. Eng..

[11] Sánchez-Badillo J.A., Gallo M., Rutiaga-Quiñones J.G., Garza J., López-Albarrán P. (2022). Insights on the cellulose pretreatment at room temperature by choline-chloride-based deep eutectic solvents: An atomistic study. Cellulose.

[12] Zhou H., Guan Y., Yan X., Pan Z., Xu J., Dai L., Zhang M., Si C. (2023). All-lignocellulose-based hard bioplastic. Ind. Crops Prod..

[13] Zhou Y., Zhang X., Cheng Y., Zhang J., Mi Q., Yin C., Wu J., Zhang J. (2022). Super-rapid and highly-efficient esterification of cellulose to achieve an accurate chromatographic analysis of its molecular weight. Carbohydr. Polym..

[14] Duan L., Liu R., Duan Y., Li Z., Li Q. (2022). A simultaneous strategy for the preparation of acetylation modified cellulose nanofiber/polypropylene composites. Carbohydr. Polym..

[15] Lei W., Fang C., Zhou X., Li Y., Pu M. (2018). Polyurethane elastomer composites reinforced with waste natural cellulosic fibers from office paper in thermal properties. Carbohydr. Polym..

[16] Yuan T., Du W., Bai K., Huang D., Nguyen T.T., Li J., Ji X. (2022). Preparation of an environment-friendly fiberboard with high mechanical strength using delignified wood fiber. Vacuum.

[17] Wu Y., Yang L., Zhou J., Yang F., Huang Q., Cai Y. (2020). Softened Wood Treated by Deep Eutectic Solvents. ACS Omega.

[18] Collard F.-X., Blin J. (2014). A review on pyrolysis of biomass constituents: Mechanisms and composition of the products obtained from the conversion of cellulose, hemicelluloses and lignin. Renew. Sustain. Energy Rev..

[19] Özsin G., Pütün A.E. (2017). Insights into pyrolysis and co-pyrolysis of biomass and polystyrene: Thermochemical behaviors, kinetics and evolved gas analysis. Energy Convers. Manag..

[20] Song J., Chen C., Zhu S., Zhu M., Dai J., Ray U., Li Y., Kuang Y., Li Y., Quispe N. (2018). Processing bulk natural wood into a high-performance structural material. Nature.

[21] Zia F., Zia K.M., Nazli Z.-H., Tabasum S., Khosa M.K., Zuber M. (2020). Preparation of hydroxyethyl cellulose/halloysite nanotubes graft polylactic acid-based polyurethane bionanocomposites. Int. J. Biol. Macromol..

[22] Zuber M., Shah S.A.A., Jamil T., Asghar M.I. (2014). Performance behavior of modified cellulosic fabrics using polyurethane acrylate copolymer. Int. J. Biol. Macromol..

[23] Hou D.-F., Liu Z.-Y., Zhou L., Tan H., Yang W., Yang M.-B. (2020). A facile strategy towards heterogeneous preparation of thermoplastic cellulose grafted polyurethane from amorphous regenerated cellulose paste. Int. J. Biol. Macromol..

[24] Yao X., Qi X., He Y., Tan D., Chen F., Fu Q. (2014). Simultaneous Reinforcing and Toughening of Polyurethane via Grafting on the Surface of Microfibrillated Cellulose. ACS Appl. Mater. Interfaces.

[25] Zhang J., Zhang X., Li M.-C., Dong J., Lee S., Cheng H.N., Lei T., Wu Q. (2019). Cellulose nanocrystal driven microphase separated nanocomposites: Enhanced mechanical performance and nanostructured morphology. Int. J. Biol. Macromol..

[26] Çetin N.S., Özmen N., Birinci E. (2011). Acetylation of Wood with Various Catalysts. J. Wood Chem. Technol..

[27] Tserki V., Zafeiropoulos N.E., Simon F., Panayiotou C. (2005). A study of the effect of acetylation and propionylation surface treatments on natural fibres. Compos. Part A Appl. Sci. Manuf..

[28] Hill C.A.S., Khalil H.P.S.A., Hale M.D. (1998). A study of the potential of acetylation to improve the properties of plant fibres. Ind. Crops Prod..

[29] Zafeiropoulos N.E., Williams D.R., Baillie C.A., Matthews F.L. (2002). Engineering and characterisation of the interface in flax fibre/polypropylene composite materials. Part I. Development and investigation of surface treatments. Compos. Part A Appl. Sci. Manuf..

[30] Colom X., Carrasco F., Pagès P., Cañavate J. (2003). Effects of different treatments on the interface of HDPE/lignocellulosic fiber composites. Compos. Sci. Technol..

[31] Lepetit A., Drolet R., Tolnai B., Zerrouki R., Montplaisir D. (2017). Effect of acetylation on the properties of microfibrillated cellulose-LDPE composites. J. Appl. Polym. Sci..

[32] de Souza F.M., Kahol P.K., Gupta R.K. (2021). Introduction to Polyurethane Chemistry. Polyurethane Chemistry: Renewable Polyols and Isocyanates.

[33] Liu N., Zhao Y., Kang M., Wang J., Wang X., Feng Y., Yin N., Li Q. (2015). The effects of the molecular weight and structure of polycarbonatediols on the properties of waterborne polyurethanes. Prog. Org. Coat..

[34] Cakić S.M., Ristić I.S., Marinović-Cincović M., Špírková M. (2013). The effects of the structure and molecular weight of the macrodiol on the properties polyurethane anionic adhesives. Int. J. Adhes. Adhes..

[35] Banerjee S.S., Aher N., Patil R., Khandare J. (2012). Poly(ethylene glycol)-Prodrug Conjugates: Concept, Design, and Applications. J. Drug Deliv..

[36] Rhodes A., Sandhu S.S., Onis S.J., Williams R. (2011). 2—Surface modification of biomaterials by covalent binding of poly(ethylene glycol) (PEG). Surface Modification of Biomaterials.

[37] Nogales A., Mitchell G.R., Vaughan A.S. (2003). Anisotropic crystallization in polypropylene induced by deformation of a nucleating agent network. Macromolecules.

[38] Bartczak Z., Lezak E. (2005). Evolution of lamellar orientation and crystalline texture of various polyethylenes and ethylene-based copolymers in plane-strain compression. Polymer.

[39] Olszewski A., Kosmela P., Piszczyk Ł. (2024). Towards sustainable catalyst-free biomass-based polyurethane-wood composites (PU-WC): From valorization and liquefaction to future generation of biocomposites. J. Clean. Prod..

[40] Srivabut C. (2024). Multi-objective optimization of turning process parameters and wood sawdust contents using response surface methodology for the minimized surface roughness of recycled plastic/wood sawdust composites. Compos. Part C Open Access.

[41] Yang A., Zhang R., Xu Z., Liu T., Fang Y., Wang W., Xu M., Song Y., Wang Q. (2024). Preparation of situ microfiber-reinforced co-extruded high-filled wood-plastic composite with excellent mechanical, creep resistance, and water resistance properties. Constr. Build. Mater..

[42] Zhang J., Xia L., Fu Z., Sun X., Zhou S., Liu X., Zhang C., Xu W. (2022). Fabrication of polyurethane porous composite films using biomass-based Juncus effususus fibers for oil removal from water. Ind. Crops Prod..

[43] Gosselink R.J.A., Snijder M.H.B., Kranenbarg A., Keijsers E.R.P., de Jong E., Stigsson L.L. (2004). Characterisation and application of NovaFiber lignin. Ind. Crops Prod..

[44] Wang Y.-Y., Wang Y., Zhu W., Lan D., Song Y.-M. (2023). Flexible poly(butylene adipate-co-butylene terephthalate) enabled high-performance polylactide/wood fiber biocomposite foam. Ind. Crops Prod..

[45] Tai N.L., Adhikari R., Shanks R., Adhikari B. (2017). Starch-polyurethane films synthesized using polyethylene glycol-isocyanate (PEG-iso): Effects of molecular weight, crystallinity, and composition of PEG-iso on physiochemical characteristics and hydrophobicity of the films. Food Packag. Shelf Life.

[46] Olszewski A., Kosmela P., Piszczyk Ł. (2023). A novel approach in wood waste utilization for manufacturing of catalyst-free polyurethane-wood composites (PU-WC). Sustain. Mater. Technol..

[47] Liu Y., Zhu W., Li Z., Xin R., He Y., Yang J., Li S., Chen M. (2024). Bamboo-based cellulose nanofibers as reinforcement for polyurethane imitation wood. Ind. Crops Prod..

[48] Wang Y.-Y., Li M., Wyman C.E., Cai C.M., Ragauskas A.J. (2018). Fast Fractionation of Technical Lignins by Organic Cosolvents. ACS Sustain. Chem. Eng..

[49] Fanta G.F., Felker F.C., Hay W.T., Selling G.W. (2017). Increased water resistance of paper treated with amylose-fatty ammonium salt inclusion complexes. Ind. Crops Prod..

[50] (2022). Standard Test Method for Measurement of the Surface Tension of Solid Coatings, Substrates and Pigments Using Contact Angle Measurements.

[51] Iyer K.A., Flores A.M., Torkelson J.M. (2015). Comparison of polyolefin biocomposites prepared with waste cardboard, microcrystalline cellulose, and cellulose nanocrystals via solid-state shear pulverization. Polymer.

[52] Prambauer M., Paulik C., Burgstaller C. (2015). The influence of paper type on the properties of structural paper—Polypropylene composites. Compos. Part A Appl. Sci. Manuf..

[53] Masoodi R., Pillai K.M. (2012). A study on moisture absorption and swelling in bio-based jute-epoxy composites. J. Reinf. Plast. Compos..

[54] Gao J., Wang X., Xu Q., Kuai B., Wang Z., Cai L., Ge S., Zhang Y.L., Li G. (2023). Efficient preparation and properties of wood fiber transparent materials with powdered wood. Ind. Crops Prod..

[55] Tran M.H., Phan D.-P., Lee E.Y. (2021). Review on lignin modifications toward natural UV protection ingredient for lignin-based sunscreens. Green Chem..

[56] Liu T., Liu Z., Zhou Z., Shi S.Q., Aladejana J.T., Gong S., Fang Z., Li J. (2023). A novel sol-gel strategy for constructing wood fibers and aramid nanofiber nanocomposite with strong, tough and recyclable properties. Compos. Sci. Technol..

[57] Wu Z., Zhang X., Kang S., Liu Y., Bushra R., Guo J., Zhu W., Khan M.R., Jin Y., Song J. (2023). Preparation of biodegradable recycled fiber composite film using lignin-based polyurethane emulsion as strength agent. Ind. Crops Prod..

[58] Ning Y., Deng H.-H., Li Q.-M., Mi Q.-H., Han B., Ai X.-Z. (2015). Effects of red and blue light quality on the metabolites and key enzyme activities of carbon-nitrogen metabolism in celery. Zhiwu Shengli Xuebao Plant Physiol. J..

